# The Lymphatic System in Zebrafish Heart Development, Regeneration and Disease Modeling

**DOI:** 10.3390/jcdd8020021

**Published:** 2021-02-19

**Authors:** Xidi Feng, Stanislao Travisano, Caroline A. Pearson, Ching-Ling Lien, Michael R. M. Harrison

**Affiliations:** 1 The Saban Research Institute of Children’s Hospital Los Angeles, Los Angeles, CA 90027, USA; xidifeng@usc.edu (X.F.); stravisano@chla.usc.edu (S.T.); 2 Laboratory of Neurogenetics and Development, Brain and Mind Research Institute, Weill Cornell Medical College, New York, NY 10021, USA; cap4010@med.cornell.edu; 3 Department of Surgery, Keck School of Medicine, University of Southern California, Los Angeles, CA 90033, USA; 4 Department of Biochemistry & Molecular Medicine, Keck School of Medicine, University of Southern California, Los Angeles, CA 90033, USA; 5 Cardiovascular Research Institute, Weill Cornell Medical College, New York, NY 10021, USA; 6 Department of Cell and Developmental Biology, Weill Cornell Medical College, New York, NY 10021, USA

**Keywords:** cardiac lymphatic vessels, zebrafish, heart, development, regeneration

## Abstract

Heart disease remains the single largest cause of death in developed countries, and novel therapeutic interventions are desperately needed to alleviate this growing burden. The cardiac lymphatic system is the long-overlooked counterpart of the coronary blood vasculature, but its important roles in homeostasis and disease are becoming increasingly apparent. Recently, the cardiac lymphatic vasculature in zebrafish has been described and its role in supporting the potent regenerative response of zebrafish heart tissue investigated. In this review, we discuss these findings in the wider context of lymphatic development, evolution and the promise of this system to open new therapeutic avenues to treat myocardial infarction and other cardiopathologies.

## 1. Introduction

### 1.1. The Lymphatic System: Discovery and Functions

In the 5th-century BC, Hippocrates described the presence of nodes containing a milky fluid (chyle) in specific subcutaneous and deep organ regions of the body [1]. Through the gallant efforts of a collection of anatomists, including Thomas Bartholin, Olaus Rudbeck and George Joyliffe, the “lymphatic vessels (*vasa lymphatica*)” were defined [1]. However, it was not until the 18th century, with the work of Mascagni and others, that it was appreciated that these lymph-containing vessels and nodes are an integral part of a network, which extends from blind-ended lymphatic capillaries (or initial lymphatics) to collecting lymphatic vessels that eventually connect to the blood circulatory system. In comparison to the blood circulatory system, understanding the role and development of the lymphatic system has been slow. This is in part due to the network being delicate and largely invisible in comparison to the obvious sphygmic blood circulatory system. The term lymph was coined to reflect this property coming from the Greek Nymph, a creature associated with clear streams and the Roman deity Lympha, meaning spring of clear water [1]. Even with the evolution of microscopy investigation, studies on the lymphatic system remained stubbornly hindered by a paucity of good molecular markers and labels. Nonetheless, just as blood vasculature is essential for the supply of oxygen and nutrients in addition to the removal of waste, the lymphatic vasculature also provides vital support of healthy tissue. As the range of techniques and technologies to study the lymphatic vasculature continues to expand, so does our understanding of the unique and critical roles this system plays in tissue homeostasis and disease [2].

The lymphatic system provides a unidirectional conduit for the essential flow of fluid from the tissue interstitium back to the circulatory system. This fluid regulation is absolutely critical, and malformation (primary lymphedema) or disruption (secondary lymphedema) of lymphatic vessels results in disabling swelling of the tissue [3,4]. This lymph fluid is rich in plasma proteins and also contains immune cells and antigens. Through the various afferent lymphatics, lymph nodes are exposed to intact or degraded microorganisms and toxic stimuli [5]. These must be removed from the fluid before being returned to the blood flow, and recent studies suggest a population of neutrophils and macrophages in the lymphatic system prevent the systemic spread of tissue pathogens [6,7]. In addition to these innate immune cells, the lymphatic vasculature plays a critical role in supporting adaptive immune responses. Immune cells that ingest foreign antigens are brought into contact with lymphocytes in the node where they present antigens to activate the adaptive responses [5]. The lymphatic vessels are not just a passive conduit that is homogenous throughout the body, but they also have tissue-specific roles, including the active absorption of lipids and vitamins. Gut villi lymphatic vessels, called lacteals, take up dietary-fats as triglyceride particles known as chylomicrons packaged by the gut enterocytes [8]. These are then transported to the systemic blood system via collecting vessels and the thoracic duct [8]. As a result, lacteal control of lipid absorption has been implicated in obesity and its sequelae, including the impact on heart disease and function [9,10]. However, as we will discuss in this review, the lymphatic system also has an immerging direct role in supporting cardiovascular health and disease.

### 1.2. The Evolution of the Lymphatic System

Most invertebrates have an open circulatory system and no distinction between a lymphatic and blood system or their respective functions [11]. Vertebrates have a range of lymphatic system features, including lymphatic vessels, lymph nodes, lymphoid organs and tissues that appear to become increasingly distinct and specialized [12]. Jawless and cartilaginous fish lack lymphatic vessels; however, some thin-walled sinuses provide a conduit for extravascular fluid back into veins in these lower vertebrates [11]. In other vertebrates, lymphatic vasculature has contractile regions, which actively aid the flow of lymph into the venous circulation [11,13]. So-called lymph hearts have been identified in lungfish, amphibians, reptiles and some flightless birds. They are typically found at the junction between lymphatic and venous systems and have been lost in higher vertebrates [11,13]. A lymphatic system that often lacks a lymph heart similar to that of mammals is found in other species of bony fish (teleosts) [11]. However, the connection of this lymphatic system to the blood circulation appears to vary across teleosts species and organ systems. In trout and glassfish, an arterial connection to the blood system has been described, and the fluid of these secondary vasculature systems can become perfused with blood under hypoxic conditions [14]. Zebrafish have been shown to have an extensive lymphatic system throughout the body, and analyses of the zebrafish vasculature system suggest it shares many conserved anatomical features with the mammalian system [15,16]. The zebrafish system has bicuspid valves and a venous connection but lacks nodes [15,17]. The possibility that this lymphatic system also retains the ability to be perfused under extreme conditions has been contested [18,19,20,21]. Reflecting the systems increasing specialization, it is likely that a spectrum of blood and lymphatic vasculature interconnectedness exists across teleosts species. Nonetheless, the zebrafish has provided invaluable insight into the molecular regulation of lymphatic development and provides a fascinating evolutionary nexus to gain a deep understanding of lymphatic function in disease.

### 1.3. The Zebrafish Lymphatic System

The majority of research has focused on zebrafish lymphatic development during the embryonic stage, taking advantage of various transgenic and tracing tools and body transparency. Lymphangiogenesis of the trunk lymphatic, facial lymphatic and intestinal lymphatic network has been well characterized in zebrafish embryos. The lymphangioblasts of trunk lymphatic vessels are derived from the posterior cardinal vein and migrate to the dorsal myoseptum to become parachordal lymphangioblasts by 2 days post-fertilization (dpf) [16,22,23,24]. Those lymphangioblasts migrate along intersomitic arteries dorsally and ventrally, forming intersomitic lymphatic vessels. The fish trunk lymphatic vasculature continues to develop to form the thoracic duct under the dorsal aorta and the dorsal longitudinal lymphatic vessel along the dorsal longitudinal anastomotic vessel by 5 dpf [15,16].

The development of facial lymphatic vessels starts from the budding of the lymphangioblasts from the common cardinal vein forming the facial lymphatic sprout (FLS) at 36 h post fertilization (hpf) [25,26]. The FLS migrate along the primary head sinus (PHS) towards the head area. The formation of facial lymphatic vessels is not from a single source of lymphangioblasts. As the FLS migrates, lymphanigoblasts originating from the PHS and the ventral aorta join the FLS, making up a complex facial lymphatic network together.

The origin of intestinal lymphatics has not been identified. There is a large lymphatic vessel associated with the entire zebrafish intestine [26,27], indicating the intestinal lymphatics may also play a role in lipid transporting as in mammals. Unlike trunk lymphatic vessels that migrate along arteries, intestinal lymphatic vessels have been found to form along both arteries and veins. This suggests that there may be tissue-specific guidance cues that guide lymphatic endothelial cell (LEC) migration.

Although the development of the lymphatic system is well-studied in zebrafish embryos, the functional studies of lymphatic vessels in regeneration and disease models in different organs are in their infancy. In this review, we will discuss the recent work on zebrafish cardiac lymphatic vessels in heart regeneration and the implications of this for our understanding of the role of lymphatic vessels in heart disease.

## 2. The Development of the Lymphatic System

### 2.1. Venous and Non-Venous Origins

The lymphatic vasculature includes a network of LECs found in close proximity to, but separate from, the blood vasculature [28]. After carrying out ink-injection experiments in pig embryos, Florence Sabin hypothesized that the majority of lymphatic vessels bud off from the endothelium of the veins and that these primitive lymphatics then spread throughout the entire embryo body to create the lymphatic network. However, after injecting along the aorta, she also concluded that, despite budding from veins, the deep lymphatics follow arteries [29]. Cell lineage studies and grafting experiments in birds validated different sources of the lymphatic vascular system. The deeper parts of the jugular lymph sacs originate from the jugular segments of the cardinal veins and the superficial, dermal lymphatics from local lymphangioblasts in the dermatomes, while the LECs of the lymph heart is of somitic origin [30].

The first cardiac lymphatic described in the human embryo grows from two different plexuses. The first one near the left jugular lymph sac, elongating between the pulmonary trunk and the aorta and following the right coronary artery. The second plexus, described as the main one, terminates in the right jugular sac and follows the left coronary artery around embryonic week eight [31]. This is in contradiction to the mouse, in which the cardiac lymphatic vessels follow the course of the cardiac veins rather than the coronary arteries [32]. In zebrafish, the development of the cardiac lymphatics occurs during late juvenile to early adult stages after two months post-fertilization (mpf) when coronary arteries, not veins, provide a scaffold for the elongation of the lymphatic vessels and the expansion of the network [33,34]. This similarity between zebrafish and human cardiac lymphatic development could represent an ancestral mechanism of essential guidance cues for the cardiac lymphatic endothelium, which has been altered across mammalian species [35].

The lymphatic vasculature is thought to form exclusively by sprouting from embryonic veins (lymphangiogenesis). Lineage tracing experiments in mice embryos demonstrated that the lymphatic system has largely venous origins [36]. Time-lapse imaging in developing zebrafish embryos demonstrated that this process is well-conserved and that at least the main thoracic duct-like vessel arises embryonically from primitive veins [16]. However, the discovery of alternative non-venous origin(s) of LECs in mammals that contribute to the lymphatic vasculature of the skin [37], mesentery [38], and heart [32,39,40] has changed the understanding of the mechanisms of embryonic lymphatic vessel development. Furthermore, evidence of a non-venous lymphatic progenitor, named “ventral aorta lymphangioblast” (VA-L), was found to give rise to facial lymphatic in zebrafish, suggesting that the origin and development of lymphatic vessels is tissue context-dependent [41].

### 2.2. Molecular Mechanism of LEC Identity

The equilibrium between endothelial cell fate regulators, Notch, Coup-TFII (Nr2f2), and Prox1 may play a critical role in the specification of endothelial cell (EC) fate during vascular development and arteriovenous-lymphatic cell fate specification [42]. Notch signaling promotes arterial EC differentiation, while in venous ECs, Notch activity is repressed by the COUP-TFII orphan nuclear receptor to maintain the vein identity [43]. The specification of the LECs in mammals is dependent on PROX1, a key transcriptional factor also crucial for maintaining the lymphatic endothelial identity [44,45]. Transcription factors *Sox1*8 [46], *CoupTFII* [47], *Gata2* [48,49], and *Hhex* [50] have been found to regulate *Prox1* expression in mouse LECs.

LEC progenitors relocate from the cardinal vein through paracrine action of VEGF-C expressed by the neighboring mesenchyme to form the primitive lymph sacs [51,52]. LECs express VEGFR2 and VEGFR3, as well as the co-receptor neuropilin 2 (NRP2) [52,53]. It was also demonstrated that VEGF-C and VEGF-D act through VEGF receptor 3 (VEGFR-3) to induce lymphangiogenesis [54,55]. LYVE-1, one of the proteins expressed in mature LECs is also expressed in a subset of ECs from the large central veins and provides the first signal of lymphatic endothelial competence [56,57].

Similar to mammals, venous-derived lymphatic progenitors in zebrafish can be detected with *prox1a* expression [22,24], and lymphatic sprouting is reliant on *vegfr3* (known as *flt4* in zebrafish) [15,58]. However, the functionally related transcription factors Coup-TFII (Nr2f2) and Sox18 were found to be dispensable for lymphatic specification in zebrafish, suggesting that transcriptional regulation of lymphatic commitment may have diverged somewhat between zebrafish and mice [59]. However, it is not known if other Nr2f factors can compensate for the loss of Nr2f2.

## 3. The Development of Cardiac Lymphatic System in Zebrafish

A cardiac lymphatic vessel system in adult zebrafish has been identified [33,34,60] (Table 1). The zebrafish cardiac lymphatic vessels express common LEC markers discussed above, including *prox1a*, *lyve1b*, *flt4* and also *mrc1a* and *stab1* [33,34,60]. When cardiac LECs migrate, the very first 1–5 tip cells are primarily labeled by *flt4* [34]. Unlike mammals that develop their cardiac lymphatic vessels at embryonic stages, lymphatic vessels are found to develop in the zebrafish post-embryonically [33,34]. The zebrafish cardiac lymphatic vessels arise from ventral facial lymphatics, which migrate along the ventral aorta [34]. The cardiac lymphatic vessel sprouts are visible at the tip of bulbus arteriosus (BA) at 21–28 DPF before any coronary vasculature development on the zebrafish heart ventricle has occurred [33,34].

**Figure 1:**
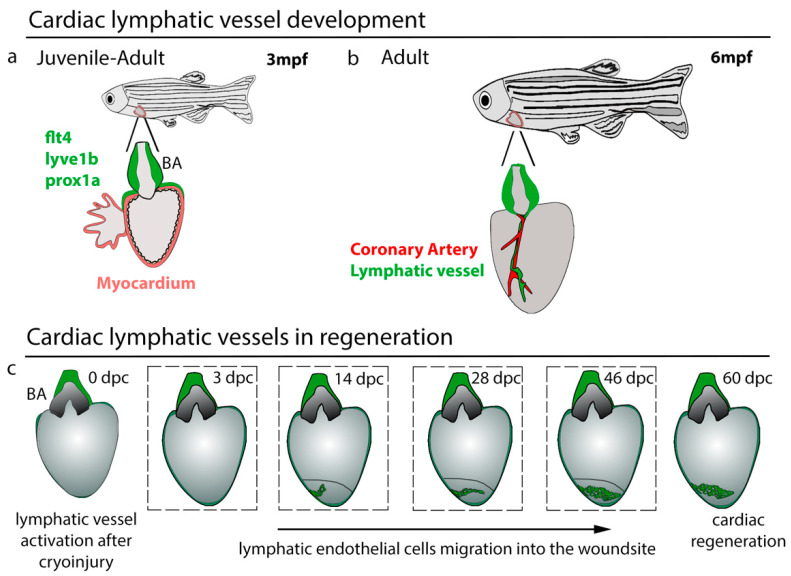
Cardiac lymphatic vessel development and regeneration. (**a**) Cardiac lymphatic vessels (*flt4*+, *lyve1b*+, *prox1a*+) that reside on bulbus arteriosus (BA) start to migrate down to the ventricle after 3 months post-fertilization (mpf) when juvenile fish mature to adults. (**b**) Cardiac lymphatic vessels follow the course of the coronary artery to populate the ventricle. (**c**) Lymphatic activation during heart regeneration. After cryoinjury, the lymphatic vasculature starts to migrate into the wound at 14 days post-cryoinjury (dpc), over the wound site and is crucial for supporting the regenerative response.

The emergence of cardiac lymphatic vessels on the heart has been shown to be correlated with the heart rate increase during the larval to the juvenile transition [34]. Reducing heart rate with the β-blocker Atenolol attenuates cardiac lymphatic sprouts on the BA and impacts the BA lymphatic branch. The BA is the fish cardiac outflow tract with a special thick-wall chamber to adjust the blood flow pressure from the fish ventricle [61]. The sprouts on the BA continue to develop and expand to form an extensive lymphatic network by eight weeks post-fertilization (wpf) [33,34]. The cardiac lymphatic vessels on the BA remain stable and do not bud from this until young adult stages when the LECs emerge onto the heart ventricle around 12–16 wpf (Figure 1a,b). These cells migrate and form vessels along the main coronary arteries, verified by *dll4*, *kdrl*, *flt1*, and *cxcr4a* expression, and also within subepicardial fat tissue [33,34]. The functional significance of this expansion into adipocytes is not known, but interestingly an upregulation of lipid metabolism genes occurs in cardiac lymphatic defective zebrafish, indicating that lymphatic vessels in zebrafish may also have a role in lipid flux in cardiac tissue [60]. Zebrafish cardiac lymphatic vessels do not appear to have open connections with the blood vasculature in resting states, as confirmed by intravascular injection [34]. However, if such connections exist and open under stress remains to be determined.

The development of zebrafish cardiac lymphatic vessels is dependent on Vegfc-Flt4 signaling [33,34,60] (Table 1). The deletion of the *flt4* receptor completely blocks the emergence of cardiac LECs on both BA and heart ventricles [34]. Since *vegfc* mutation is embryonic lethal in zebrafish, cardiac lymphatic vessel dependence on Vegfc was characterized in *vegfc* heterozygotes. The reduction of Vegfc ligand dramatically affected the lymphatic coverage and branching on BA. The sprouts and growth were reduced in the *vegfc* heterozygotes [34] (Table 1). Similar results were observed in hypomorphic *vegfc* mutants on a *vegfd* mutant background [60]. Cardiac lymphatic vessels were still detectable on the BA in zebrafish with either one functional *vegfc* or *vegfd* allele but lacking on heart ventricles of these zebrafish. In hypomorphic *vegfc* and amorphic *vegfd* double mutants, the cardiac lymphatic vessels were absent on both BA and heart ventricles [60]. In order to investigate the role of Vegfc signaling in cardiac lymphatic vessel extension in isolation of the more systemic effects on lymphatic development at earlier stages, Harrison et al. blocked the Vegfc signaling by a heat-inducible expression of soluble Flt4 (sFlt4) receptor [33]. The induction of *sflt4* after the establishment of cardiac lymphatic vessels on BA resulted in no lymphatic vessel formation on the zebrafish ventricle. This indicates that the ventricular extension of the lymphatic vessels specifically requires Vegfc signaling and addition to any prior requirement in the specification. The coronary vessels are also required for normal cardiac lymphatic vessel growth providing a scaffold that can promote the extension of the lymphatic vessels onto the ventricle [33,34]. Phenylhydrazine hydrochloride (PHZ)-induced coronary vasculature overgrowth also promoted cardiac lymphatic development in zebrafish [34]. In contrast, in *cxcr4a* mutants without normal coronary vasculature, the growth of cardiac lymphatic vessels was also blocked on the ventricles [33,34]. Notably, the VFL and cardiac lymphatic vessels on the BA did not show obvious defects in mutants, indicating that the lack of cardiac lymphatic vessels extension onto the heart ventricle is mainly due to the loss of coronary vasculature [33,34].

## 4. The Role of the Cardiac Lymphatic System in Heart Homeostasis, Disease, and Regeneration

### 4.1. Roles of Lymphatic Vessels in Cardiovascular Disease

The lymphatic vessels play a prominent role in lipid metabolism. Intestinal lymphatics take up dietary lipids in the form of lipoprotein particles known as chylomicrons to transport them to the bloodstream [62]. Furthermore, lymphatic endothelium is a passive exchange perimeter indispensable for the transport of cholesterol [63]. Although vascular smooth muscle cells are the major cell type responsible for plaque formation in murine models of atherosclerosis, contributing to almost 70% of all plaque cells [64], the hypothesis that atherosclerosis is a chronic inflammatory disease of the arterial wall has gained widespread acceptance [65]. Elevated serum cholesterol levels and hypertension are very well-known risk factors for cardiovascular disease [66]. Despite the fact that blood vessels are more frequent than lymphatics in the collagenous outside (*adventitia*) surrounding a coronary plaque, the lymphatic vessels are highly present in the inner layers (*intima* and *media*) of progressive atherosclerotic lesions of coronary arteries and their growth is associated with areas characterized by scattered calcium deposits and cholesterol crystals [67]. In addition, it has been shown that the specific blockage of the VEGFR-3 decreases lymphatic vessel activation and local cardiac inflammation after transplantation and could be used as a novel lymphatic vessel–targeted immunomodulatory therapy [68]. A better understanding of the cardiac lymphatic system may offer new possibilities for therapeutic interventions in the future.

The blockage of coronary arteries by an atherosclerotic plaque results in the death of surrounding cardiac muscle in events known as myocardial infarction (MI). The necrotic tissue will further cause acute inflammation response, edema and tissue remodeling at the infarcted site, leading to fibrotic scar, arrhythmia and eventually heart failure [69]. Recently, an increasing number of studies have demonstrated the importance of cardiac lymphatic vessels in MI. The lymphangiogenesis at the infarcted area has been observed in artery ligation induced MI mice [32] and rats [70] and in post-MI human patient samples [71]. It has been shown that cardiac lymphatic vasculature has a protective role in post-MI recovery in mice [72,73,74]. The blockage of VEGF-C signaling by soluble decoy VEGFR3 (sVEGFR3) results in impaired morphology of cardiac lymphatic vessels [72]. The survival rate in sVEGFR3 mice after MI was dramatically reduced compared to wildtype (WT) controls. Further analysis revealed an increase in scar size and intramyocardial hemorrhages in sVEGFR3 mice. Furthermore, the scar composition measured by non-invasive MRI in sVEGFR3 mice was found different from that of WT controls. Apelin (APLN), the ligand for the G-protein-coupled APJ receptor, is important for lymphatic vasculature maturation [75]. The knockout of apelin in mice affected the cell–cell junction integrity in LECs and resulted in dilated lymphatic vessels [73]. Without healthy cardiac lymphatic vasculature, apelin knockout mice suffered a more serious inflammation response after MI.

One of the important functions of lymphatic vessels is immune cell clearance at the inflammation site, which has been shown to be essential for cardiac function after MI [74]. *Lyve-1* deletion in mice did not affect the overall development of lymphatic vessels [76] but was deleterious to leukocyte docking [77]. The *Lyve-1* mutant mice with defective immune cell clearance exhibited more fibrotic tissue and reduced percentage LV ejection fraction and stroke volume in the hearts after MI [74].

Besides its physiologic function in MI, the cardiac lymphatic vessels also secrete signal molecules in regulating heart repair. Lui et al. have shown that Reelin (RELN), an extracellular matrix protein mainly expressed by cardiac LECs, regulates heart growth and promotes cardiomyocyte (CM) proliferation during development in mice hearts [78]. During heart repair in neonatal mice, *Reln* expression was highly induced at the injury site [78]. The deletion of RELN diminished the heart repair with increased scar size and reduced heart function [78]. Consistent with its role in heart development, CM proliferation was reduced and CM apoptosis elevated in *Reln* mutants after MI. Together these studies suggest cardiac lymphatic vessels have a supportive role in post-MI recovery.

**Figure 2:**
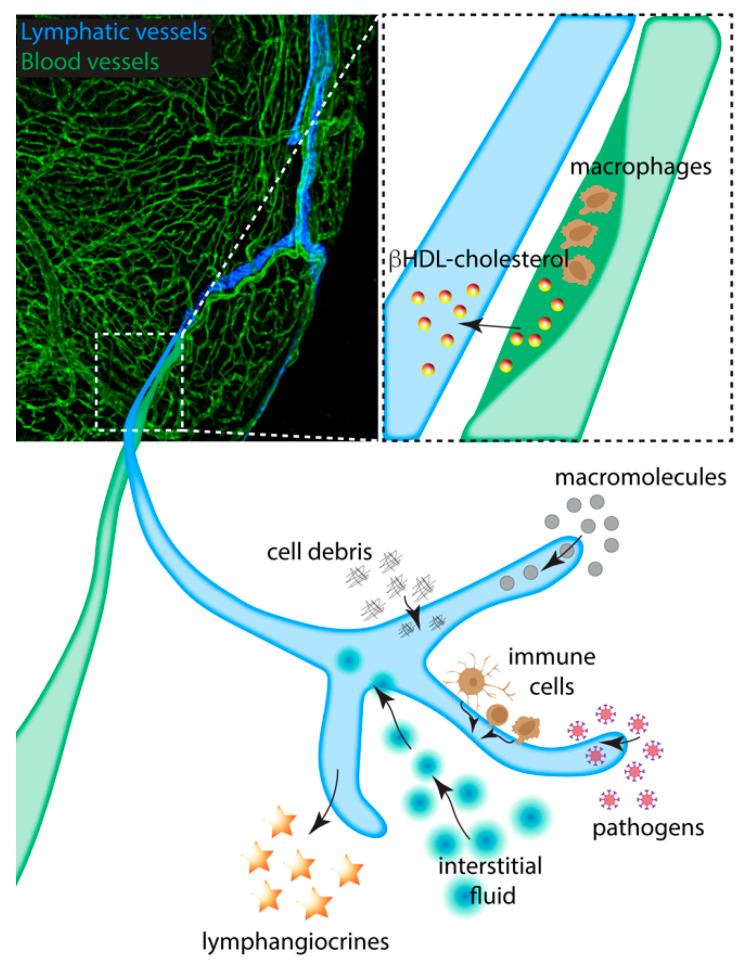
The roles of the cardiac lymphatic system in heart disease and regeneration. Coronary artery occlusion by atherosclerotic plaque causes myocardial infarction. The plaque is composed of infiltrated macrophage and cholesterol deposits; cardiac lymphatics running along the artery provide a conduit for cholesterol as a βHDL complex to be removed from the heart and returned to the liver (boxed area). During development, homeostasis and disease, the cardiac lymphatics uptake cell debris, macromolecules, immune cells, pathogens and fluid. Insufficiency of such removal can result in inflammation and edema induced fibrosis, which is detrimental for clinical outcomes. The lymphatic vasculature responds to such insufficiency by the expansion of the lymphatic capillaries after damage to the myocardium. The lymphatic endothelial cells are also a source of lymphangiocrines, excreted proteins that promote regeneration and growth of the myocardial tissue.

Therapeutic induction of cardiac lymphangiogenesis by VEGF-C appears to have a beneficial role in MI. The application of VEGF-C promoted lymphangiogenesis and improved cardiac function in both rats [70] and mice [32] after MI. In VEGF-C-treated rats, immune cell clearance was increased, and cardiac edema and collagen deposition were decreased compared to controls [70]. The potential roles of cardiac lymphatic vessels in heart disease and regeneration are summarized in Figure 2.

### 4.2. The Function of Cardiac Lymphatics in Zebrafish Heart Regeneration

Compared to mammals, zebrafish have the amazing capacity to fully regenerate heart tissue after injury, making it an ideal model to study the function of cardiac lymphatic vessels in heart regeneration [79]. Cardiac lymphatics have been shown to have distinct responses in different injury models [33,34]. After amputation, few hearts had limited cardiac lymphatic vessel growth into the wound area during heart regeneration [33,60]. The amputation has less inflammation and only minor collagen/fibrin deposition due to clean removal of the cardiac tissue. In contrast, a dramatic lymphangiogenesis response was induced in zebrafish hearts after cryoinjury, with a large number of lymphatic vessels migrating into the wound area and forming a network with increased branches and enlarged vessel diameter [33,34]. Compared to amputation, cryoinjury is a more complex heart regeneration model, which incorporates components of necrosis and inflammation, with injured tissue and ECM persisting in the wound area. A similar response occurs with injury to the fin suggesting necrotic tissue is important for neo-lymphatic growth after injury [60]. The lymphangiogenic response during heart regeneration is also regulated by Vegfc-Flt4 signaling. *Vegfc* expression in zebrafish heart became undetectable after 14 days post-amputation (dpa) while still remaining in the heart wound area after 42 days post-cryoinjury (dpc) [33]. In addition, cardiac lymphatic vessel growth was completely absent in *flt4* mutants and highly reduced in *vegfc* hets after cryoinjury [34]. Consistent with this lymphangiogenic response, cardiac lymphatic vessels also show important roles in heart regeneration after cryoinjury. In the hearts with defective cardiac lymphatic vessel development, heart regeneration after cryoinjury was also impacted compared to WT controls; this was not seen in the heart without cardiac lymphatic vessels after amputation [33,34,60]. The difference in zebrafish heart regeneration after amputation and cryoinjury suggests that cryoinjury may be a more suitable injury model to study the functions of cardiac lymphatic vessels since there are server inflammation and necrotic tissue at infarctional sites in post-MI human hearts [69].

The functions of zebrafish cardiac lymphatic vessels in cryoinjury appear to include homeostasis maintenance and immune cell clearance. The cardiac lymphatic vessels were able to absorb intramyocardial injected Qdots (<10 nm diameter) and transport *mpx*+ neutrophils recruited after cryoinjury [33]. However, *mpx*+ neutrophil clearance was attenuated in zebrafish heart without cardiac lymphatic vessels after cryoinjury [33]. Terminal deoxynucleotidyl transferase dUTP nick end labeling (TUNEL) staining, which detects DNA breaks in apoptosis, revealed an accumulation of TUNEL-positive signals at the infarcted area in cardiac lymphatic vessel impacted hearts in zebrafish [60]. These results indicate that the functions of cardiac lymphatic vessels in immune cell clearance and necrotic cell removal are essential for efficient heart regeneration after cryoinjury in zebrafish. This suggests a therapeutic benefit in targeting a patient’s cardiac lymphatic vessels after MI. According to zebrafish heart regeneration results, the induction of cardiac lymphangiogenesis after MI may prevent long-term inflammation and fibrotic scar deposition. It will be interesting to investigate further the dysregulation of myocardial metabolism in the zebrafish lacking cardiac lymphatics and potential effects on myocardial proliferation and regeneration.

## 5. Future Directions

The zebrafish is an emerging model to study development, regeneration, and model human disease due to their amenability for imaging and available forward and reverse genetic tools. The studies of trunk lymphatic vessels in zebrafish embryos have provided valuable insights into lymphatic development. Different organs, including the heart, may utilize organ/tissue-specific mechanisms to regulate fluid homeostasis and immune cell modulation to accommodate their physiological demands, and this is currently under intense study. For the roles of cardiac lymphatic vessels, the following aspects can be further clarified and studied.

### 5.1. Cardiac Lymphatic Formation and Populations

We and others have performed a detailed characterization of cardiac lymphatic vessel development and neo-lymphangiogenesis during zebrafish heart regeneration as a basis for future studies. One unexpected aspect of the cardiac lymphatic vessels in zebrafish is their discontinuous nature over the ventricle. A conduit is formed as observed with Qdot uptake following intramyocardial injection [33], but also individual or small groups of LECs were often observed in connection with the main cardiac lymphatic vessel or isolated from it [33,34,60]. Interestingly, Gancz et al. found that this population has a different sensitivity to signaling changes suggesting that isolated cells may not require the scaffold of the coronary arteries. Furthermore, additional signaling pathways and sources may be directing cardiac lymphatic development. Understanding the development of cardiac lymphatic vessels at the cellular level and the signaling that shapes them will be critical to therapeutically encourage (or discourage) their formation.

It remains unclear whether these isolated lymphatic cells and clusters are truly a distinct population or if they are an artifact of the formation of this delicate lymphatic vessel. They may reciprocally dissociate and associate from the main vessel as it expands during development and regeneration. This is consistent with the observed reduced sensitivity of isolated LECs to loss of *cxcr4a*, which manifests as a range of phenotypic severity [33,80]. In *cxcr4a* mutant zebrafish that develop some coronary vasculature, this may be sufficient to support limited LEC outgrowth and expansion but still insufficient for complete vessel formation. Significantly, these isolated LEC clusters were transiently observed in mouse embryonic hearts, but the origins of the clusters were found to be indistinguishable from the main vessel [34]. This suggests that the LEC clusters could be derived from the main lymphatic vessel in a process that may be similar to that observed during lung development [81]. The clusters are transient in the mouse, not being identifiable at later stages. As the development of the cardiac vessel progresses, these clusters may progressively fuse with the main vessel.

Regardless of origin, it is also possible that isolated LEC populations can contribute to heart regeneration. They appear in zebrafish heart during regeneration after cryoinjury [34]. Furthermore, the identification of the first lymphangiocrine, RELN, suggests that the positive benefits post-MI are not limited to the lymphatics acting as a conduit in the classical sense [78]. Individual cells could excrete pro-regenerative factors or provide scavenger functions much like those described of brain LECs/fluorescent granular perithelial cells [82,83]. It will be fascinating to further uncover the unexpected support functions and morphogenic events of LECs in developmental and regenerative contexts.

### 5.2. Signaling Pathways Regulating Cardiac Lymphatic Vessel Expansion

Many different signaling pathways emanating from coronary vasculature or otherwise might be further explored. One candidate signaling pathway is Notch, which is known to regulate EC proliferation, motility, filopodia formation, adhesion, and vessel stabilization [84]. Notch receptors and ligands such as Notch1 and Dll4 are predominantly expressed in arterial endothelial cells during embryonic development and arterial cell specification [85,86]. Activation of Notch 1 by Dll4-positive venous ECs (VECs) has been shown to induce a lymphatic transcription profile and so transcriptional activation of Notch signaling may be required to reprogram VEC into LEC [87]. Moreover, genetic targeting of Notch impaired LEC migration during embryonic zebrafish development [87] and blocking its activation by *Dll4*-expression leads to downregulation of *Lyve1* and *EphrinB2* both in vitro [42] and in vivo [88]. Conversely, the lack of Notch activity resulted also in enhanced lymphatic sprouting leading to an increased LEC proliferation/survival in mice [89,90]. The role of Notch signaling in cardiac LECs is less well understood and will require further study.

### 5.3. Role of Cardiac Lymphatics in Myocardial Infarction

A key role of lymphatic vasculature is the clearance of interstitial fluid. Loss of cardiac lymphatic vessels on the ventricle did not appear to give rise to overt interstitial edema [33,34,60]. Only with loss of *vegfd* together with compromised Vegfc function was hypertrophy observed, but it is not clear if this is caused by interstitial edema. In most conditions of compromised Vegfc-Flt4 and/or coronary vessel signaling, the BA lymphatic vessels remain largely unaffected, and this may be sufficient to provide a conduit for fluid removal. It remains to be determined if the hypertrophy observed in the *vegfc* hypermorph; *vegfd* double mutant (*vegfc^hy−/−^; vegfd^−/−^*) is due to loss of the BA populations, misregulation of Flt4/Vegfr3-independent signaling or a compensatory effect of earlier reductions in cardiomyocyte proliferation due to loss of mitogens as observed in the mouse [78]. The phenotypic variability observed with the *vegfc^hy−/−^; vegfd^−/−^* combination, indeed all the variability in reported phenotypes across the three studies using various mutant alleles and reporters, needs to be considered in light of varying modifiers in the genetic background [91].

The damage to heart tissue that occurs in response to MI is complex, involving hypoxia, necrosis, inflammation and fibrosis. The cryoinjury model of zebrafish heart incorporates these features more robustly than the amputation injury. Complexity in a model can occlude analysis of specific processes; however, the cost of this simplicity is that not all features of the regenerative response are captured with the amputation model. In amputation, there is a lack of lymphangiogenesis, and the regenerative response is not perturbed with loss of lymphatics on the ventricle, both, however, are observed after cryoinjury [33,34,60]. Comparison of the models provides a useful insight into which processes are driving the expansion of lymphatic vessels, their roles at the wound site and how these can be utilized to resolve the complex post-MI environment observed in patients.

## Figures and Tables

**Table 1:** Summary of the genetic tools and findings of papers describing the zebrafish cardiac lymphatic system.

	Vivien et al. [60]	Harrison et al. [33]	Gancz et al. [34]
Cardiac lymphatic vessels (LVs)	*prox1a^+^ lyve1b^+^*	*prox1a^+^ flt4^+^ lyve1b^+^ (high BA; low ventricle) mrc1a^+^ stab1^+^*	*prox1a^+^ flt4^+^ lyve1b^+^ mrc1a^+^*
LVs in regeneration (cryoinjury)	*prox1a^+^*	*prox1a^+^ flt4^+^ lyve1b^+^(low) mrc1a^+^*	*prox1a^+^ flt4^+^ lyve1b^+^ mrc1a^+^*
Roles of LVs	Cardiac hypertrophy, metabolic homeostasis, and inflammation resolution	Cardiac regeneration, cell debris clearance and inflammation resolution	Cardiac regeneration
Mutants lacking BA LECs	*vegfc^hy^* *^−^* *^/^* *^−^* *; vegfd* *^−^* *^/^* *^−^*	-	*flt4* *^−^* *^/^* *^−^*
Mutants/Tg lacking ventricular LECs	*vegfc^hy+/−^; vegfd^−/−^* *vegfc^hy^* *^−^* *^/^* *^−^* *; vegfd^+/−^*	*sFlt4* *cxcr4a^−/−^ (majority)*	*vegfc^+/−^, flt4^−/−^* *cxcr4a^−/−^ (isolated LECs unaffected)*
Mutants with hypertrophy	*vegfc^hy^* *^−^* *^/^* *^−^* *; vegfd* *^−^* *^/^* *^−^*	-	-
Mutants/Tg defective scar resolution	*vegfc^hy^^−^^/^^−^; vegfd^−^^/^^−^* (30%)	*cxcr4a^−/−^* *sFlt4*	*vegfc^hy−/−^; vegfd^−/−^, flt4^−/−^* *cxcr4a* *^−^* *^/^* *^−^*

## Data Availability

Data sharing not applicable.
